# Stabilization of the Bio-Oil Organic Phase via Solvent-Assisted Hydrotreating, Part 1: Investigating the Influence of Various Solvents

**DOI:** 10.3390/bioengineering12050537

**Published:** 2025-05-16

**Authors:** Manqoba Shezi, Manish Sakhakarmy, Sushil Adhikari, Sammy Lewis Kiambi

**Affiliations:** 1 Department of Chemical Engineering, Durban University of Technology, Durban 4000, South Africa; 2 Department of Biosystems Engineering, Auburn University, 200 Corley Building, Auburn, AL 36849, USA; 3 ASI Industrial, 1300 Minnesota Ave, Billings, MT 59101, USA; sakhakarmym@gmail.com; 4 Chemical Engineering Department, Vaal University of Technology, Vanderbijlpark 1900, South Africa

**Keywords:** biomass, bio-oil stabilization, solvents, mild hydrodeoxygenation, dehydration

## Abstract

Conventional mild hydrotreatment processes of bio-oil present significant challenges of a high degree of polymerization, a low oil yield, high coke formation, and poor catalyst recovery. To address these challenges, the current study looked into investigating and enhancing the properties of raw bio-oil organic phase samples via a solvent-assisted stabilization approach using methanol (METH), ethanol (ETH), isopropyl alcohol (IPA), and ethyl ether (DME). Solvents like methanol (METH) and ethanol (ETH), which are highly polar, yielded higher oil fractions (64% and 62%, respectively) compared to less polar solvents like ethyl ether (DME) at 59%. Isopropyl alcohol (IPA), with intermediate polarity, achieved a balanced oil yield of 63%, indicating its ability to dissolve both polar and non-polar components. Moisture reduction in stabilized bio-oils followed the order IPA > ETH > METH > DME, with IPA showing the highest reduction due to its structural characteristics facilitating dehydration. Viscosity reduction varied, with IPA > ETH > DME > METH. Carbon recovery in stabilized bio-oils ranged from 65% to 75% for DME, ETH, and METH and was 71% for IPA. The heating values of stabilized bio-oils ranged from 28 to 29 MJ/kg, with IPA-stabilized bio-oil showing the highest value (29.05 ± 0.06 MJ/kg). METH demonstrated high efficiency (74.8%) in stabilizing bio-oil, attributed to its strong hydrogen-donating capability. ETH followed closely at 69.5%, indicating its comparable performance in bio-oil stabilization. With moderate efficiency (69.3%), IPA presents a balanced alternative considering its molecular structure and hydrogen solubility. In contrast, DME exhibited lower efficiency (63.6%) due to its weaker hydrogenation capability and propensity for undesired side reactions. The current study suggests that subcritical conditions up to 200 °C are adequate for METH, ETH, and IPA in bio-oil stabilization, comparable to results obtained under supercritical conditions.

## 1. Introduction

Biomass, among other alternative energy sources, has become a focal point due to global concerns surrounding diminishing fossil fuel supplies, rapid population expansion, growing energy requirements, and the instability of crude oil prices. Biomass can be a renewable resource for producing gaseous, liquid, and solid biofuels [1].

The thermochemical processing of biomass is gaining traction as a viable route for producing petrochemicals and biofuels. This approach often utilizes second-generation feedstocks, which are rich in lignin and cellulose and sourced from non-food resources like forest residues. These feedstocks present a sustainable advantage over first-generation options by mitigating potential impacts on food security [2]. Within thermochemical conversion, several key techniques—pyrolysis, liquefaction, gasification, and combustion—are employed to transform various biomass materials into numerous products, demonstrating significant productive capacity [3].

Pyrolysis stands out among the thermochemical methods described earlier due to its cost-effectiveness and simplicity in converting biomass into biofuels and petrochemicals. This technique is classified into three categories: slow, fast, and flash pyrolysis [4]. The desired product distribution dictates the selection of pyrolysis method. For maximizing bio-oil output, fast pyrolysis is preferred. Conversely, slow and flash pyrolysis are more effective for generating biochar and pyrolysis gas, respectively. The bio-oil produced through fast pyrolysis holds promise as a source of valuable petrochemicals. However, due to undesired properties, it is incompatible with direct application as an engine fuel [5]. The low heating value and high water content, acidity, and corrosiveness are well-known limitations of raw bio-oil. The aforementioned drawbacks of raw bio-oil are attributed to the presence of oxygenated compounds such as phenols, alcohols, furans, water, acids, ketones, and aldehydes [6,7]. Consequently, eliminating oxygenates is adopted to improve the quality of raw bio-oil [8].

Conventional upgrading technologies include hydrotreatment, catalytic cracking, and steam reforming, with the former being the most efficient due to the high yields of fuel-range hydrocarbons [6,9]. Furthermore, the hydrotreating process has already been commercialized in crude oil refineries to upgrade the distillates. Thus, bio-oil upgrading via hydrotreating is economically and commercially viable. Researchers have explored numerous hydrotreating catalysts globally. These catalysts include sulfided metals (NiMo, CoMo, and NiW), reduced noble (Pg, Pt, and Ru), and transition (Co, Fe, Mo, Ni, and Cu) metals with selected support carriers [10,11,12,13]. The well-known supports are alumina-based (Al_2_O_3_), silica-based (SiO_2_), titanium-based (SiO_2_), and carbon-based (C) supports [12,14,15,16]. An alumina-based support is considered unsuitable for application during the hydrotreatment of raw bio-oil due to the formation of boehmite (AlO(OH)), which is yielded due to the high water content in raw bio-oil [11]. Hence, a neutral support such as carbon is a convenient option during the hydrotreatment of raw bio-oil [14].

Noble metal-based catalysts have higher catalytic activity than transition metal catalysts for hydrotreating, thus providing simplified operating conditions [17,18]. Metal sulfide catalysts have also proven their robust industrial application for upgrading heavy petroleum fractions, with bimetallic phases, such as sulfide NiMo, being more effective when compared to monometallic due to the synergy effect [12]. Nonetheless, sulfided metal catalysts (NiMo and CoMo) have a significant potential drawback of deactivation due to the low sulfur content in raw bio-oil [11]. The hydrotreatment process for upgrading bio-oil has been extensively studied and shown to effectively reduce oxygenates, thereby enhancing the quality and stability of bio-oil [13].

Conventionally, hydrotreatment of bio-oil is carried out in two stages, with the first stage (mild conditions) serving to stabilize the bio-oil and the second stage (severe conditions) upgrading the bio-oil by increasing the energy density via the elimination of oxygenates and the reduction in moisture content. However, during the first stage, there are still drawbacks, such as polymerization, a low oil yield, and poor catalyst recovery. Hence, during the second stage (350–450 °C, 5–15 MPa), significant gas and char formation as by-products are observed due to the aforementioned drawbacks of the conventional mild hydrotreatment process. This has spurred research efforts toward developing hydrotreatment processes that eliminate polymerization and poor catalyst recovery while enhancing bio-oil properties. Hence, in this work, a solvent-assisted bio-oil stabilization technique has been carried out to address the drawbacks of the conventional first-stage hydrotreatment process. Methanol (METH), ethyl ether (DME), isopropyl alcohol (IPA), and ethanol (ETH) are common solvents with distinct properties. METH (polar, bp 64.7 °C) and ETH (bp 78.4 °C) are water-miscible and polar-protic, whereas IPA (bp 82.6 °C) is less polar. DME (nonpolar, bp 34.6 °C) is highly volatile and flammable. METH is toxic, while ETH is safer for biological applications. All have low viscosities (<2.04 mPa·s) and densities (~0.7–0.8 g/mL).

The main goal of adopting the solvent-assisted stabilizing technique of bio-oil is to eliminate compounds responsible for coking (aldehydes and furans) and reduce the degree of polymerization. Although oxygen is undesired in the treated product, completely removing oxygen from bio-oil is very difficult, especially under mild conditions. Hence, this study focused more on the properties of the stabilized bio-oil, namely density, viscosity, total acid number (TAN), elemental analysis, the degree of deoxygenation, the degree of dehydration, moisture content reduction, and energy efficiency.

## 2. Materials and Methods

### 2.1. Materials

A commercially available catalyst, Ru/C, procured from Sigma-Aldrich (St. Louis, MO, USA), was employed in this study. The catalyst contained 5 wt% metal loading, had a BET surface area of 686.55 m^2^/g and a pore diameter of 3.3 nm, and was utilized without any prior treatment, i.e., used as received. The solvents used in the bio-oil stabilization experiments included methanol (anhydrous, ≥99.8%, CAS: 67-56-1) and isopropyl alcohol (≥99.5% ACS grade, CAS: 67-63-0), procured from VWR (Radnor, PA, USA), as well as ethanol (absolute, ≥99.5% ACS, 200 proof, CAS: 64-17-5) and ethyl ether (anhydrous, 99+%, stabilized, ACS grade, CAS: 60-29-7), supplied by Thermo Scientific Chemicals (Thermo Fisher Scientific, Waltham, MA, USA). All solvents were used as received without further purification, given their high purity and compliance with ACS/analytical specifications. The bio-oil organic phase was produced from the thermal degradation of pinewood biomass in a fluidized bed reactor.

### 2.2. Fast Pyrolysis Procedure

The fast pyrolysis method employed in this study was previously described in detail by Shezi and Kiambi [19]. Briefly, pinewood biomass (7.75 ± 0.19 kg) was subjected to fast pyrolysis in a fluidized bed reactor at 544 ± 33 °C, using silica sand as a fluidizing medium. Following separation from the non-condensable gases, the bio-oil organic phase (BOP) was collected continuously in pre-weighed Nalgene bottles (Sigma-Aldrich, Style 2104, Cat. Nos. B9157 and 89407). To maintain the integrity of the bio-oil, which comprises a vast array of organic compounds, the bottles were promptly sealed airtight and transferred to a dark cold room maintained at 4 °C. This storage method was chosen to minimize light-induced reactions and chemical transformations that could occur in the bio-oil, thereby preserving its quality for subsequent hydrotreating experiments. For a complete description of the reactor setup, operating conditions, and product collection procedures, see Shezi and Kiambi [19]. In the current study, we investigated the impact of various solvents on the mild hydrotreating process, which was not addressed in the previous work.

### 2.3. BOP Stabilization Procedure

**Figure 1:**
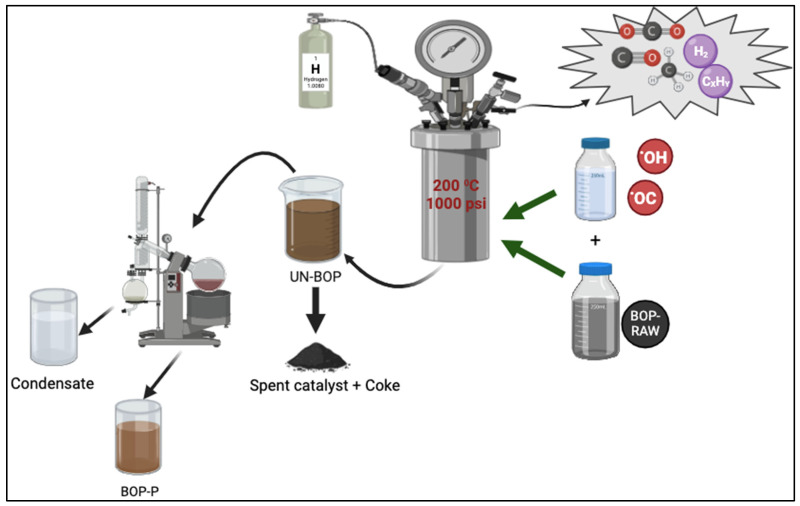
Schematic flow of solvent-assisted bio-oil organic phase stabilization.

This investigation explored the stabilization (mild hydrodeoxygenation) of bio-oil using various solvents. The schematic flow of the solvent-assisted approach is shown in Figure 1. The first step was blending bio-oil with each solvent to obtain a feed to the stabilization system. The BOP–solvent feed blends were prepared by taking an 80% by-weight sample of BOP and mixing it with 20% by-weight of solvent in a glass container. The mixture was placed onto a vortex mixer for 3 ± 1 min to achieve homogeneity. The feed blends and the desired amount of catalyst were transferred into the 450 mL stirring Parr reactor (Parr Instrument Company, Moline, IL, USA). The feed-to-catalyst ratio was 70:1. The reactor was sealed cautiously to avoid damaging the nuts. Following reactor sealing, a leak test was conducted under 1500 psi of hydrogen with a holding time of 4 min. The leak test was performed three times to incorporate sufficient system purging. Upon confirming no leaks, hydrogen was released to displace residual air, and the reactor was vacuumed. The reactor was pressurized to 1000 psi for the stabilization experiments, and the temperature was ramped up to the set point with stirring at 500 rpm. The set-point temperature was 200 °C, with a retention time of 4 h, exclusive of heating. After each experimental run, the reactor was allowed to cool down to room temperature. The final pressure was recorded, and the gas sample was collected using a 1 L Tedlar bag (RESTEK, Bellefonte, PA, USA). The final mass of the reactor with stabilized oil was weighed. Liquid products were then collected, and catalyst particles were separated via centrifugation. Coke products and used catalysts were recovered via vacuum filtration and rinsed with feed solvent. The solid-free liquid product was separated using a rotary evaporator to obtain the stabilized bio-oil. The percentage yields after bio-oil stabilization were calculated by the following equations:

(1) $$\mathrm{Liquid}\;\mathrm{Yield}=\frac{\mathrm{Liquid}\;\mathrm{product}(g)}{\mathrm{BOP}\left(g\right)+\mathrm{Solvent}(g)}\times 100$$

(2) $$\mathrm{Solids}\;\mathrm{Yield}=\frac{\mathrm{Solids}\;\mathrm{product}(g)}{\mathrm{BOP}\left(g\right)+\mathrm{Solvent}(g)}\times 100$$

(3) Gas Yield=100−liquid yield−solids yield

The degree of deoxygenation (DOD) and degree of dehydration (DOD^h^) were determined by the following equations:

(4) $$\mathrm{DOD}=(1-\frac{\mathrm{Oxygen}\;\mathrm{in}\;\mathrm{product}}{\mathrm{Oxygen}\;\mathrm{in}\;\mathrm{feed}})\times 100$$

(5) $${\mathrm{DOD}}^{h}=(1-\frac{{\mathrm{MC}}_{P}}{{\mathrm{MC}}_{F}})\times 100$$

(6) $$\frac{H}{C_{eff}}=\frac{H}{C}-2\left(\frac{O}{C}\right)$$

where MC_P_ and MC_F_ are the moisture contents for stabilized bio-oil (BOP-P) and feed bio-oil (BOP-Raw), respectively. The ratio of H/C_eff_ is the effective carbon ratio that incorporates the removal of oxygen in the form of water.

### 2.4. Product Characterization

The carbon, hydrogen, nitrogen, and sulfur content of the samples was determined following ASTM D5373 [20] and ASTM D5291 [21] using an Elementar Vario Micro Select analyzer (Langenselbold, Germany). Oxygen content was calculated by subtracting the CHNS values from 100%, expressing the result on a dry basis. Water content in the bio-oil was assessed via Karl Fischer titration, employing Aquastar (Geneva, Switzerland) as the reagent/titrant and Apura (Coral Gables, FL, USA) as the solvent. For this volumetric Karl Fischer titration, the reagent was “combititrant 5 keto” (targeting aldehydes and ketones), and the solvent was “combi-solvent keto” (containing approximately 5 mg H_2_O). Acidity was quantified by measuring the total acid number (TAN) according to ASTM D664-07 [22]. A Mettler T50 autotitrator (Karl Fisher, Mettler Toledo, Columbus, OH, USA) was used for this purpose, with a total acid number titration solvent mixture and 0.1 M KOH 2-propanol serving as the titrant. Kinematic and dynamic viscosity, along with density, were measured at 40 °C using an Anton Paar instrument, following the device’s standard method (SVM). The chemical composition of the bio-oil’s organic phase was determined through gas chromatography–mass spectrometry (GC-MS). Analysis was performed on an Agilent 5977C GC/MSD system (Santa Clara, CA, USA), utilizing helium as the carrier gas. Compound identification relied upon the National Institute of Standards and Technology (NIST) spectral library. Samples were prepared by dilution with 2 mL of methanol before injection into the GC-MS. The inlet temperature was set at 280 °C with a 10:1 split ratio. Chromatographic separation was achieved using a temperature program that held the column at 50 °C for 5 min, followed by a ramp to 280 °C at 10 °C/min and a final hold of 5 min. To investigate carbon deposition on the spent catalyst, thermogravimetric analysis was conducted. A TG-50H detector (SHIMADZU Thermogravimetric Analyzer, Long Beach, CA, USA) equipped with an alumina cell was employed, operating under an air atmosphere (20 mL/min) with a heating rate of 10 °C/min to 800 °C, where the temperature was maintained for 2 min.

## 3. Results and Discussion

### 3.1. Product Yields

**Figure 2:**
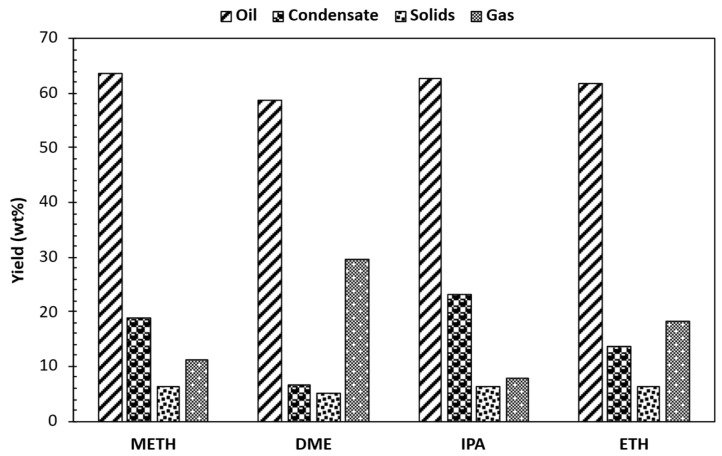
Product distribution of solvent-assisted bio-oil organic phase stabilization.

The overall mass balance closure for liquid and solid components reached 80.90%, 90.75%, and 91.37% following the reactions with METH, ETH, and IPA, respectively (as shown in Figure 2). The solids incorporated tar; thus, crystallization was necessary. Filtration, solvent washing, and drying were adopted for crystallization to quantify solid yields. The tar loss was negligible, representing less than 1% of the total stabilized bio-oil yield. The results of the bio-oil stabilization process with different solvents reveal distinct trends in product yields. Solvents with higher polarity, such as METH and ETH, demonstrate enhanced oil yields of 64% and 62%, respectively, compared to less polar solvents like DME with a yield of 59%. Hence, DME, possessing lower polarity, yielded a marginally lower oil fraction, suggesting a reduced affinity for polar compounds. This disparity reflects the ability of polar solvents to effectively solubilize polar compounds present in the bio-oil during solvent-assisted mild hydrotreatment. IPA, with intermediate polarity, yields an oil fraction of 63%, suggesting a balanced ability to dissolve both polar and non-polar components. Conversely, DME yields the highest gas percentage at 30%, indicative of its propensity for vigorous hydrogenation reactions, followed by ETH at 18%. The observed gas yield trend was DME > ETH > METH > IPA. The complex process of bio-oil upgrading involves a variety of chemical transformations. These transformations, which include cracking, decarboxylation, decarbonylation, methanation, and hydrodenitrogenation, contribute to the production of gaseous products [23]. The observed increase in gas yield suggests that more of the DME bio-oil underwent decomposition into gas products compared to METH, IPA, and ETH bio-oils. These results indicate a stronger propensity for DME, relative to METH and IPA, to induce the fragmentation of higher-molecular-weight fractions in the bio-oil and the evolution of gaseous species during the stabilization process. The greater reactivity of DME may have been responsible for the observed increase in mass losses, potentially a consequence of the enhanced volatility of the products formed. Moreover, the possibility of solvent self-decomposition under supercritical conditions contributing to a portion of the gaseous products cannot be discounted [24]. Isopropyl alcohol, with its intermediate polarity, stands out for its elevated condensate yield of 23%, compared to the other solvents, the observed trend was IPA > METH > ETH > DME. This suggests its potential to promote the formation of water molecules during mild hydrotreatment. The observed high condensate yield for IPA correlated with the highest moisture content reduction of 8%. The solids yield remained relatively consistent across solvents, ranging from 5% to 6%, indicative of the robustness of the solvent-assisted hydrotreating process. The actual amount of solids is much less compared to the one depicted in Figure 2, which incorporated tar.

### 3.2. Physicochemical Properties of Raw, Blended, and Stabilized BOP

The properties of treated bio-oil, such as thermal stability, acidity, and heating value, were enhanced by dehydration, hydrocracking, hydrogenation, and mild deoxygenation during the stabilization of bio-oil. The solvent can also act as a co-reactant, as indicated by variations in proximate and ultimate analysis (Table 1). After blending with each solvent, the properties of the raw bio-oil varied. As a result, bio-oil stabilization is highly affected by the choice of solvent used. The current study explores how the solvent affects bio-oil stability and quality; this was carried out by investing the differences in properties arising from the inclusion of different solvents during the solvent-assisted mild hydrotreatment process. The investigated parameters were density, viscosity, total acid number (TAN), elemental analysis, the degree of deoxygenation, moisture content reduction, and energy efficiency. Since solvent addition affects raw properties, blending variations were also incorporated.

**Figure 3:**
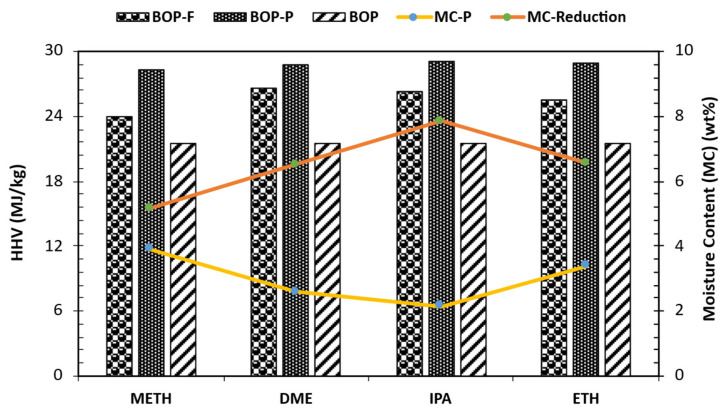
Energy density and moisture content reduction of stabilized bio-oil organic phase.

Table 1 summarizes the findings from analyses, including water content, density, viscosity, heating value, TAN, and CHNS. The bio-oil feed blends of METH and DME resulted in a reduction in moisture content in contrast to then IPA and ETH feed blends. This phenomenon agrees with the observation made by Yu et al. [25] on the METH feed blend. However, it differs for IPA and ETH feed blends. Yu et al. [25] noted that blending BOP with methanol, ethanol, and isopropanol led to a decrease in moisture content. Pidtasang et al. [26] suggested that the reduction in moisture levels is a consequence of dilution by anhydrous alcohols. Hence, the observed increases in moisture were attributed to the possible hydrous effect of IPA and METH. The increased moisture observed in METH and ETH bio-oils post solvent-assisted stabilization can be attributed to the generation of water via esterification. This reaction involves the solvents and the acidic components of the bio-oil. These results were confirmed by the GCMS analysis for METH and ETH-stabilized bio-oils. Similarly, the observed acid number for all stabilized bio-oils was subsequently lower when compared to the raw bio-oil. The primary mechanism behind this reduction in acidity and subsequent pH improvement was attributed to ester formation, oligomer repolymerization, solvent hydrodeoxygenation, and hydrocracking during the stabilization process. This resultant water was lowest for DME, as supported by the observed low condensate yield (Figure 2). The lower gas yield (Figure 2) in bio-oil IPA indicates that IPA did not decompose to gas, keeping water content relatively low. The removal of water from bio-oil is necessary due to the adverse effects of moisture on ignition delay and engine combustion performance [27]. However, water in bio-oil can be advantageous by reducing viscosity. The order of reduction in moisture content after bio-oil stabilization is IPA > ETH > METH > DME, as seen in Figure 3. The stabilized oil fractions were relatively dry, containing 2 to 4 wt% moisture. The stabilized bio-oil products exhibited lower kinematic viscosity with relatively consistent densities compared to the raw bio-oil, with no noticeable alteration in color. The viscosity ranged from 4.82 ± 1.65 to 15.18 ± 1.50 mm^2^/s for METH and IPA bio-oils, respectively. The order of decrease in viscosity was IPA > ETH > DME > METH.

### 3.3. Energy Density and Moisture Content Reduction

The energy output of the raw bio-oil was 21.58 ± 0.06 MJ/kg. Improving this output is vital for better combustion efficiency in engines. Figure 3 illustrates minor changes in energy output after the stabilization of bio-oils compared to their respective blends, as well as moisture content reduction. In the current study, the stabilization reactions do not significantly enhance the energy output compared to blending, although there is a significant drop in moisture content. The rise in energy output between the feed blends and the raw bio-oil correlated with the solvent/oil ratio of 1:6. The observed increase in energy outputs between blends and raw bio-oil was around 20% for all solvents. The results of the energy outputs indicate highly oxygenated compounds in the stabilized bio-oil, which is also evident in the GCMS and CHNS/O analyses. Increasing carbon (C) and hydrogen (H) levels while decreasing oxygen (O) results in higher energy density [28]. The DOD values were between 19 and 30%, which correlated with the observed heating values. High oxygen content in the bio-oil decreases its energy output due to reduced available carbon, which facilitates combustion. The stabilized bio-oils had heating values of 28–29 MJ/kg, with the METH bio-oil having the lowest heating value of 28.23 ± 0.04 MJ/kg. The highest HHV was obtained for IPA-stabilized bio-oil with a 29.05 ± 0.06 MJ/kg value. The energy outputs of the blends and stabilized bio-oils increased, corresponding to the energy content of the added solvents, with DME, IPA, and ETH showing the highest values due to superior properties over METH. Although the energy content of stabilized bio-oils is enhanced, it does not reach the levels of traditional fuels. Crude oil, for instance, has an energy density of 45.54 MJ/kg, and gasoline measures at 46.54 MJ/kg, both of which are higher than the values achieved by stabilized bio-oils. Nonetheless, the improvements in energy output compared to the raw bio-oil suggest that solvent-assisted bio-oil stabilization is an effective method for enhancing bio-oil properties while eliminating the coking effect due to polymerization. Moreover, this study aimed to stabilize bio-oil by improving its quality before severe treatment that potentially leads to the formation of fuel-rich hydrocarbons; thus, the observed results of heating values align with the goals of the current study.

### 3.4. Degree of Deoxygenation and Carbon Recovery

**Figure 4:**
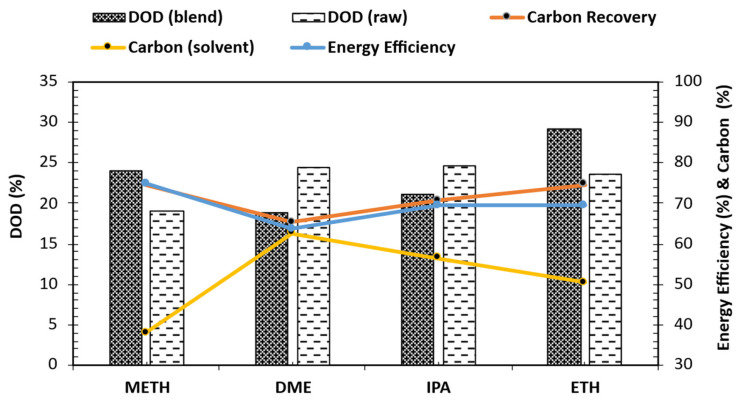
Degree of deoxygenation, carbon recovery, and energy efficiency of stabilized bio-oil organic phase [carbon (solvent) is available carbon in feed solvent].

The elemental analysis determined the mass fractions of carbon (C), hydrogen (H), nitrogen (N), and sulfur (S) present in the sample. Oxygen content (O) was calculated by difference. The data presented in Table 1 reveal a slight increase in C, H, and N contents for all stabilized bio-oils. The carbon retention/recovery for stabilized bio-oils ranged from 65 to 75% for DME, ETH, and METH, while it was 71% for IPA, as seen in Figure 4. The oxygen content was moderately lower after stabilizing the bio-oil; however, it was an anticipated outcome because mild hydrotreatment focuses more on stabilizing the bio-oil and converting compounds to corresponding alcohols, mainly xylenols. In addition to alcohol conversion, solvent addition promotes the generation of ketones. Hence, the high oxygen content was attributed to the presence of oxygenated compounds such as ketones and phenol derivatives (such as xylenols) in the stabilized bio-oils.

The observed xylenol isomers in the stabilized bio-oil were attributed to the application of the Ru/C catalyst, which promotes isomerization [29]. With all that being said, there was a reduction in oxygen after stabilizing the bio-oils. The calculated results of the DOD relative to the feed blend (DOD blend) and raw bio-oil (DOD raw) are shown in Figure 4. Employing DME, IPA, and ETH as the solvents for stabilizing the bio-oil resulted in a DOD of 25%, while METH resulted in 19% relative to the raw bio-oil. Conversely, relative to the feed blend, ETH and METH resulted in a DOD of 29 and 24%, respectively. Meanwhile, for DME and IPA, the DOD was 19 and 21%, respectively, relative to the feed blend. Available carbon in the solvent is negatively correlated to the recovered carbon in the stabilized bio-oils, as seen in Figure 4. The DME solvent has more available carbon; however, DME bio-oil carbon recovery is the lowest, which is indicative that most carbon is recovered in the gas phase due to the absence of hydroxyl groups that facilitate dehydration reactions and a high degree of volatility. The conversion of carbon to solid and gaseous products may occur through polymerization reactions and processes including decarboxylation, decarbonylation, and methanation [30,31]. Examining the energy efficiency results of treated bio-oils using various solvents during bio-oil stabilization reveals insights into the process dynamics. METH, with its high efficiency rating of 74.8%, showcases its efficacy as a stabilizing solvent. Its strong hydrogen-donating capability facilitates effective hydrogenation reactions, enhancing conversion rates and reducing energy consumption. ETH closely follows at 69.5%, owing to its comparable hydrogenation potential and conducive chemical interactions during bio-oil stabilization. METH and ETH efficiency indicate their suitability for solvent-assisted bio-oil stabilization processes. On the other hand, IPA, despite its moderate efficiency of 69.3%, presents a viable alternative, offering a balance between performance and cost considerations, potentially influenced by its molecular structure and hydrogen solubility. Conversely, DME’s lower efficiency of 63.6% hints at limitations stemming from its weaker hydrogenation capability (due to the absence of the -OH group) and propensity for undesired side reactions. The results highlight the significance of solvent polarity and reactivity profiles in dictating the energy efficiency of stabilized bio-oils.

### 3.5. GCMS Analysis of Stabilized BOP

**Figure 5:**
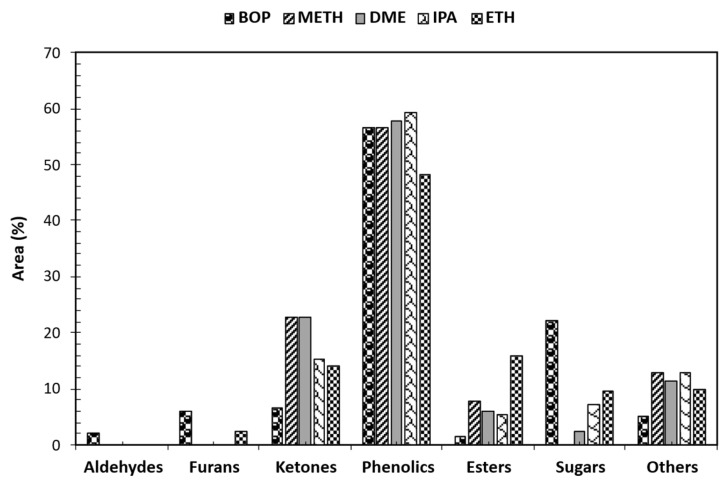
GCMS analysis of raw and stabilized bio-oil organic phase using various solvents. [BOP—raw bio-oil organic phase].

Gas chromatography–mass spectrometry (GC-MS) was used to identify and measure the quantity of different molecules present in both raw and stabilized bio-oil samples. To understand how product distribution varied across samples, the identified compounds were classified into six categories based on their functional groups: phenols, esters, ketones, aldehydes, sugar derivatives, and furans. The relative abundance of each compound class in raw and treated bio-oil is illustrated in Figure 5. The total proportional area (%) for each group was calculated by adding the individual proportional areas (%) of the compounds within that group. Because a compound’s chromatographic peak area (%) is directly related to its concentration, comparisons of peak areas can reveal changes in concentration. For example, changes in phenol concentration after each reaction can be assessed by comparing phenol peak areas (%), providing insight into the effects of solvents during bio-oil stabilization. Furthermore, the relative content of a compound within the detected compounds can be evaluated using its peak area (%).

Analyses of GC-MS for the raw bio-oil (BOP) compared to stabilized bio-oils using various solvents indicated distinct variations in compound composition expressed as area percentages. Notably, aldehydes are present in BOP (2%) but absent in all stabilized bio-oils, indicating efficient removal during solvent-assisted stabilization. Similarly, furans are notably reduced in the stabilized bio-oils, with residual furans observed only in the ETH-treated bio-oil (3%). Ketones exhibited significant variation among the samples, with the highest concentration observed in the METH and DME-treated bio-oils (23%), followed by IPA (15%) and ETH (14%). This suggests solvent-dependent effects on ketone formation or preservation during bio-oil stabilization. Phenolics were significantly present in the stabilized bio-oils, with concentrations ranging from 48 to 59%. However, the ETH stabilized bio-oil reduced phenolics and formed more esters. The abundance of phenolics after stabilization was attributed to phenolics being converted to their corresponding derivatives. The analysis revealed a significant decrease in the concentration of phenols containing unsaturated (double) bonds within their substituted groups, exemplified by 2-methoxy-4-(1-propenyl)-phenol, in the raw bio-oil after hydrotreatment. Conversely, phenols with saturated substituted groups, such as 2-methoxy-4-propyl-phenol, showed an increase. This change is consistent with the expected reduction in double bonds as a result of the hydrotreating process [32]. The observed increase in the proportion of 2-methoxy-4-propyl-phenol after the reactions suggests that it is a product of the conversion of 4-hydroxy-2-methoxycinnamaldehyde. The latter compound was not detected following bio-oil stabilization, implying that it had been transformed. Furthermore, the rise in methoxy-phenolic compounds after stabilization was attributed to the breakdown of the complex lignin structures present in the bio-oil [33]. Removal or conversion of aldehydes in bio-oil is desirable due to their role in thermal instability and carbonaceous deposit formation. Aldehydes like 5-hydroxymethyl-2-furancarboxaldehyde (HMF), prevalent in raw bio-oil, were undetectable after bio-oil stabilization. The absence of sugars in METH-treated bio-oil and the varying concentrations in other stabilized bio-oils indicate selective removal or conversion of sugars during solvent-assisted bio-oil stabilization. These findings highlight the efficacy of solvent-assisted bio-oil stabilization in reducing oxygenates and the effects on bio-oil composition. Such insights are crucial before severe hydrotreating of stabilized bio-oil to obtain hydrocarbon-rich products with diverse applications, ranging from biofuels to specialty chemicals. Compounds identified in the raw bio-oil comprise furfural, 2-methoxy-5-methyl-phenol, 1,2-Cyclopentanedione, D-Allose, 2-Furancarboxaldehyde, 5-methyl-, Benzenepropanol, 4-hydroxy-3-methoxy-, methacrylic acid, and ethyl ester. The raw bio-oil was primarily composed of phenolic derivatives (57%), which are products of lignin decomposition. Notable components included 2-Methoxy-5-methylphenol, 1,2-Benzenediol, 4-methyl-, 2-Methoxy-4-vinylphenol, trans-Isoeugenol, and 2-methylphenol. Although peak area percentage is not a quantitative measure of compound concentration, the high proportion of phenolic peak areas supports the conclusion that these compounds are abundant in crude bio-oil, a finding corroborated by other research [34,35,36,37].

**Figure 6:**
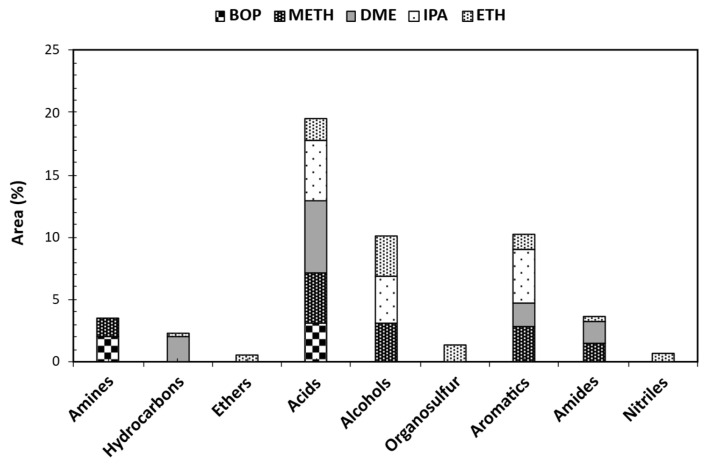
“Others” GCMS analysis of raw and stabilized bio-oil organic phase using various solvents. [BOP—raw bio-oil organic phase].

Solvent-assisted bio-oil stabilization led to a significant rise in the number of identified esters and their proportional representation within the bio-oil. Ester prevalence is preferable in fuel composition over acids due to their reduced corrosive impact on engine surfaces. Esters can form via esterification reactions between bio-oil acids and corresponding alcohols (METH/ETH/IPA). Ester formation can also occur through reactions between alcohol solvents and acids generated from intermediate products during the stabilization process. Solvents, like methanol and ethanol, have been shown to disrupt oligomer chains in crude bio-oil, leading to lower-molecular-weight compounds. The GCMS analysis in this study confirms this phenomenon. The ester product distribution was altered after the bio-oil reacted with each alcohol solvent. ETH-stabilized bio-oil showed reduced acid compounds (Figure 6), which correlated with the observed high ester presence in Figure 5. The METH, ETH, and IPA-stabilized bio-oils resulted in Propanoic acid methyl ester, 9-Octadecenoic acid, methyl ester, Heptadecanoic acid, 16-methyl-, methyl ester, Butanoic acid, 3-methyl-2-oxo-, methyl ester, and Hexadecanoic acid, methyl ester; Pentanoic acid, 4-oxo-, ethyl ester, Propanoic acid, 2-hydroxy-, ethyl ester, Butanoic acid, ethyl ester, Butanedioic acid, diethyl ester, Propanoic acid, 2-methyl-, 2-methyl propyl ester, Methacrylic acid, ethyl ester, Octadecanoic acid, ethyl ester, and Ethyl Oleate; and Pentanoic acid, 4-oxo-, 1-methyl ethyl ester, and Propanoic acid, 2-methyl-, propyl ester, respectively. DME-stabilized bio-oil resulted in nitrogen- and sulfur-containing compounds. These compounds are undesirable, and stabilizing the bio-oil also aims at removing these unwanted compounds. Aside from the undesired compounds, DME-stabilized bio-oil also yielded esters (1,2-Ethanediol, monoacetate, Sulfurous acid, 2-pentyl pentyl ester, and 1,2-Propanediol, 1-acetate). However, esterification was not prominent when compared to METH, ETH, and IPA stabilization reactions. The observed order of ester detection in the stabilized bio-oils is ETH > METH > IPA > DME. The results indicate that solvent-assisted bio-oil stabilization using methanol, ethanol, and isopropanol can facilitate ester formation.

### 3.6. Van Krevelen Plot of Raw, Blended, and Stabilized BOP

**Figure 7:**
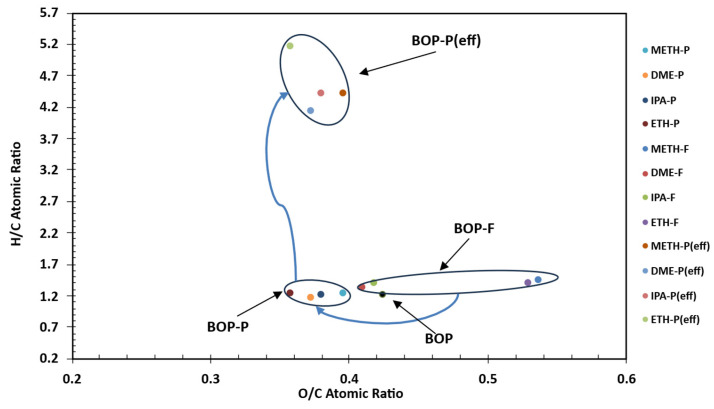
Van Krevelen plot of raw, blended, and stabilized bio-oil organic phase. [H/C_eff_ = H/C − 2(O/C), raw bio-oil (BOP), feed blends (-F), and stabilized/effective bio-oils (-P/eff)].

Figure 7 depicts a Van Krevelen plot illustrating the raw bio-oil (BOP), feed blends (-F), and stabilized/effective bio-oils (-P/eff). The graph reveals notable differences in elemental compositions and O/C ratios between the feed blends, with METH closely correlated to ETH. However, the O/C ratios in the stabilized bio-oils (BOP-P) from all experiments consistently fall within a narrow range of 0.36 to 0.40, significantly lower than those of the corresponding feed blends (BOP-F), while BOP was slightly higher, at 0.43. The solvent-assisted stabilization approach produced bio-oils with reduced O/C ratios and consistent H/C ratios relative to the raw bio-oil. Meanwhile, the METH and ETH feed blends resulted in higher O/C ratios and slight increases in H/C ratios. Conversely, DME and IPA feed blends had lower O/C ratios relative to the raw bio-oil. Higher H/C ratios are desirable for biofuel production; thus, the lower H/C ratios in the treated bio-oils do not indicate ineffective solvent-assisted stabilization. To assess the effectiveness of the solvent-assisted approach, the effective H/C ratio (H/C_eff_) was adopted, which considers the removal of oxygen content in the form of water. This metric offers a superior evaluation of the chemical structure transformation, indicating that all experiments notably enhanced the H/C_eff_ value within a narrow range of 4.1 to 5.1, implying effective stabilization of raw bio-oil. The increase in the H/C_eff_ ratio suggests dehydration reactions. These reactions are negatively correlated with the H/C_eff_ ratio, hence the observed consistency in the H/C_eff_ ratios. A decrease in the O/C ratio is indicative of bio-oil deoxygenation (oxygen reduction). From these results, it is evident that dehydration reactions were highly favored during the stabilization of bio-oil, with the ETH solvent resulting in lower O/C and higher H/C_eff_ ratios when compared to other solvents.

### 3.7. Statistical Comparison of Treatment Effects

Statistical *t*-tests (n = 3) were conducted to compare each solvent blend before and after MILD-HDO, as well as with the raw BOP. The following trends were consistently significant:

Moisture Content: All HDO-treated blends had significantly lower moisture compared to their untreated counterparts (*p* < 0.001 for DME, IPA, ETH), except for methanol (*p* = 0.063), likely due to residual hydrophilic methanol interactions [25].

Kinematic Viscosity: Viscosity reductions were statistically significant for all fuels except methanol (*p* = 0.063). The IPA-HDO blend achieved a reduction from 29.8 to 15.18 mm^2^/s (*p* = 0.0016), enhancing its fuel compatibility.

Total Acid Number (TAN): HDO treatment led to significant TAN reductions across all blends (*p* < 0.01), indicating effective removal of carboxylic and phenolic groups that contribute to corrosiveness and polymerization.

Higher Heating Value (HHV): All HDO-treated fuels exhibited statistically significant increases in the HHV (*p* < 0.0001), reflecting the combined effects of oxygen removal and reduced moisture [28]. IPA-HDO showed the highest HHV at 29.05 MJ/kg.

### 3.8. Degree of Dehydration and Deoxygenation

The degree of deoxygenation (DOD) and dehydration (DOD^h^) are critical indicators of upgrading efficiency. DOD blend values post HDO increased significantly across all solvents (*p* < 1 × 10^−6^), suggesting effective oxygen elimination. The DOD^h^ values, reflecting the loss of hydroxyl groups via dehydration reactions, showed dramatic improvements, particularly in IPA-HDO (from 1.74% to 78.46%, *p* ≈ 5.7 × 10^−10^). Among the solvents tested, IPA emerged as the most effective in enhancing HDO performance, based on the statistically significant improvements in all key biofuel properties. This synergy may stem from favorable hydrogen-donating properties and solvent–bio-oil interactions that facilitate catalytic reactions.

### 3.9. Implications for Bio-Oil Upgrading

The substantial physicochemical improvements observed in the MILD-HDO-treated BOP blends demonstrate the viability of this approach for producing fuel-compatible bio-oils. Enhanced energy density, reduced acidity, and improved fluidity all contribute to better storage stability, engine compatibility, and overall fuel quality [27]. The findings support the integration of MILD-HDO in future biorefinery designs, especially with the use of tailored solvents such as IPA or DME for process intensification.

These results provide critical evidence that upgrading lignocellulosic bio-oils using solvent-aided MILD-HDO can bridge the gap between crude pyrolysis oils and transportation-grade fuels.

### 3.10. Turnover Frequency Thermogravimetric Analysis of Catalyst

**Figure 8:**
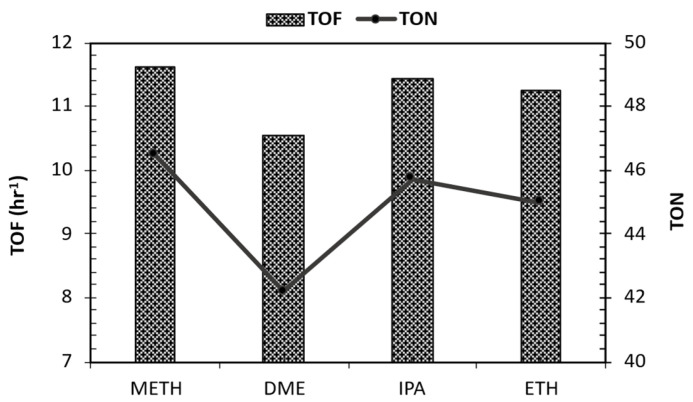
Catalyst turnover frequency during solvent-assisted bio-oil organic phase stabilization.

The analysis of turnover number (TON) and turnover frequency (TOF) data for Ru/C catalysts in the presence of different solvents during bio-oil stabilization reveals interesting insights into the catalytic performance (Figure 8). The TON values obtained for METH (46), DME (42), IPA (46), and ETH (45) indicate that these solvents contribute to almost comparable (with minimal variations) levels of catalytic efficiency in terms of product formation per gram of catalyst before deactivation. The lowest TON was observed after DME stabilization. The absence of hydroxyl groups on the DME solvent may have contributed to a lower TON due to reduced solubility. This suggests that the choice of solvent may not significantly impact the overall catalytic activity of the Ru/C catalyst during bio-oil stabilization; however, DME was undesirable for bio-oil stabilization. Additionally, the calculated TOF values, which were derived from the TON values at a reaction time of 4 h, further support this observation, with METH exhibiting the highest TOF (11.6) and DME showing the lowest TOF (10.5). These findings suggest that while the solvents may have subtle effects on the kinetics of the stabilization reactions, they do not significantly alter the overall efficiency of the catalytic process, as indicated by a 0 to 8.7% absolute error relative to the highest value.

**Figure 9:**
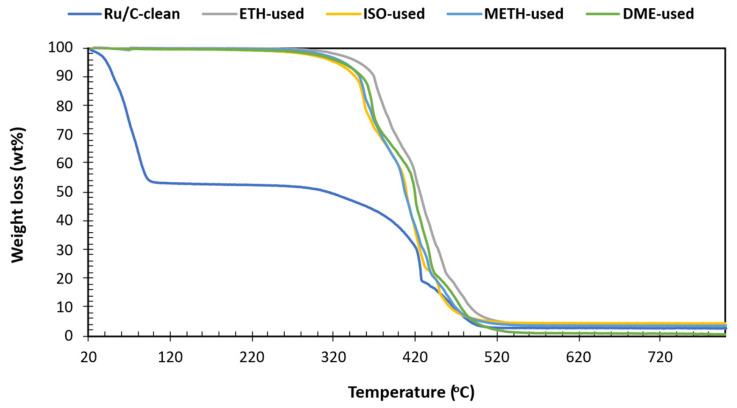
Thermogravimetric analysis of clean and spent catalyst after solvent-assisted bio-oil organic phase stabilization.

Adopting solvent-assisted bio-oil stabilization provides several advantages for minimizing carbon deposition over conventional mild hydrotreatment processes (Figure 9). The formation of coke, a generally unwanted by-product in bio-oil hydrocracking and hydrotreatment, is typically attributed to the re-polymerization and over-dehydration of oligomers [24,38]. Figure 9 shows that the profiles were closely correlated, suggesting similar carbon deposits on spent catalysts. This was also supported by the comparable solids yield post bio-oil stabilization reactions. Although the degradation profiles are almost equivalent, DME showed a high degree of coking, as indicated by the highest mass degradation of 98%. The significant increase in weight loss for DME is attributed to the combustion of the extra carbon deposited on the catalyst surface. The high degree of coking for DME is attributed to the absence of the OH group and its high volatility. The elemental analysis of solids shows a carbon content of 95–98 wt% and supports the prospect of carbon deposition via coke formation. Among the significant advantages of adopting solvent-assisted bio-oil stabilization, coke reduction, depolymerization via enhanced solubility, improved mass transfer, and dilution effect are prominent. Furthermore, the successful stabilization of intermediates (aldehydes, furans, and carboxylic acids) suppressed carbon deposition prospects for ETH, METH, and ISO.

## 4. Conclusions

The current study investigated how different solvents influence the stability and quality of bio-oil. By employing solvents with varying polarities during solvent-assisted mild hydrotreatment, significant differences in oil yields and properties were observed compared to conventional methods. Solvents like METH and ETH, which are highly polar, yielded higher oil fractions (64% and 62%, respectively) compared to less polar solvents like DME at 59%. This difference underscores the polar solvents’ ability to effectively solubilize polar compounds in bio-oil. IPA, with intermediate polarity, achieved a balanced oil yield of 63%, indicating its ability to dissolve both polar and non-polar components. The moisture reduction in stabilized bio-oils followed the order IPA > ETH > METH > DME. IPA exhibited the greatest reduction because its molecular structure promotes dehydration more effectively than the other compounds. The stabilized bio-oils exhibited lower viscosity and consistent densities compared to raw bio-oil without significant color alteration. Viscosity reduction varied, with IPA > ETH > DME > METH. Carbon recovery in stabilized bio-oils ranged from 65% to 75% for DME, ETH, and METH and was 71% for IPA. The heating values of stabilized bio-oils ranged from 28 to 29 MJ/kg, with IPA-stabilized bio-oil showing the highest value (29.05 ± 0.06 MJ/kg). The rise in energy output between the feed blends and the raw bio-oil correlated with the solvent/oil ratio of 1:6. The results of energy outputs were indicative of highly oxygenated compounds in the stabilized bio-oil, which was also evident in the GCMS and CHNS/O analyses. Aldehydes like 5-hydroxymethyl-2-furancarboxaldehyde (HMF), prevalent in raw bio-oil, were undetectable after bio-oil stabilization. Energy outputs correlated with the energy content of added solvents, with DME, IPA, and ETH providing higher values than METH due to superior properties. METH demonstrated high efficiency (74.8%) in stabilizing bio-oil, attributed to its strong hydrogen-donating capability. ETH followed closely at 69.5%, indicating its comparable performance in bio-oil stabilization. IPA, with moderate efficiency (69.3%), presented a balanced alternative considering its molecular structure and hydrogen solubility. In contrast, DME exhibited lower efficiency (63.6%) due to its weaker hydrogenation capability and propensity for undesired side reactions. This study suggests that subcritical conditions up to 200 °C are adequate for METH, ETH, and IPA in bio-oil stabilization, comparable to results obtained under supercritical conditions. Stabilization reactions further improve bio-oil properties through processes like esterification and hydrogenation, enhancing physicochemical characteristics such as heating value and viscosity. Future research should focus on optimizing solvent recovery and reuse to improve the efficiency of bio-oil stabilization processes. This approach ensures sustainability and maximizes the economic viability of bio-oil upgrading by minimizing resource consumption and waste generation.

## Figures and Tables

**Table 1:** Physicochemical properties of raw, blended, and stabilized bio-oil organic phase.

Parameters	BOP Blends				BOP-MILD-HDO				BOP
	METH	DME	IPA	ETH	METH	DME	IPA	ETH	
Proximate Analysis (wt%)									
Moisture Content ^b^	9.08 ± 0.36	9.09 ± 0.00	9.96 ± 0.03	9.94 ± 0.02	3.91 ± 0.02	2.60 ± 0.01	2.15 ± 0.23	3.38 ± 0.04	9.79 ± 0.180
Ultimate Analysis (wt%) ^db^									
Carbon	59.41 ± 0.69	64.83 ± 0.26	64.07 ± 0.74	59.83 ± 2.61	65.78 ± 0.01	67.12 ± 0.02	66.57 ± 0.15	67.44 ± 0.04	64.54 ± 0.45
Hydrogen	8.50 ± 0.12	8.50 ± 0.02	8.91 ± 0.19	8.30 ± 0.40	8.10 ± 0.01	7.77 ± 0.01	7.99 ± 0.00	8.30 ± 0.02	7.820 ± 0.02
Nitrogen	0.18 ± 0.03	0.09 ± 0.01	0.16 ± 0.02	0.16 ± 0.01	0.08 ± 0.00	0.07 ± 0.01	0.08 ± 0.01	0.06 ± 0.01	0.180 ± 0.07
Sulfur	0	0	0	0	0	0	0.04 ± 0.03	0.05 ± 0.07	0.040 ± 0.02
Oxygen ^a^	31.9 ± 0.84	26.58 ± 0.24	26.85 ± 0.95	31.71 ± 3.00	26.04 ± 0.01	25.04 ± 0.02	25.32 ± 0.11	24.16 ± 0.14	27.42 ± 0.55
C/H ratio	6.99 ± 0.02	7.63 ± 0.01	7.19 ± 0.07	7.21 ± 0.03	8.12 ± 0.01	8.64 ± 0.01	8.33 ± 0.02	8.13 ± 0.02	8.252 ± 0.82
H/C ratio	0.143 ± 0.00	0.131 ± 0.00	0.139 ± 0.00	0.139 ± 0.00	0.123 ± 0.00	0.116 ± 0.00	0.120 ± 0.00	0.123 ± 0.00	0.121 ± 0.01
O/C	0.54 ± 0.00	0.41 ± 0.00	0.42 ± 0.01	0.53 ± 0.03	0.40 ± 0.00	0.37 ± 0.00	0.38 ± 0.00	0.36 ± 0.00	0.425 ± 0.43
Density (g/cm^3^, 40 °C) ^ar^	1.09 ± 0.00	1.08 ± 0.00	1.09 ± 0.00	1.08 ± 0.00	1.13 ± 0.00	1.14 ± 0.00	1.13 ± 0.00	1.13 ± 0.00	1.18 ± 0.00
K. Viscosity (mm^2^/s, 40 °C) ^ar^	13.0 ± 1.14	13.3 ± 1.25	29.8 ± 1.77	19.9 ± 1.55	4.82 ± 1.65	5.90 ± 1.70	15.18 ± 1.50	12.25 ± 1.25	67.85 ± 1.25
D. Viscosity (mPa.s) ^ar^	14.15 ± 1.34	14.63 ± 1.50	32.32 ± 2.02	21.59 ± 1.75	5.45 ± 1.85	6.71 ± 1.95	17.16 ± 1.75	13.85 ± 1.45	80.20 ± 1.50
TAN (mgKOH/g) ^ar^	62.55 ± 2.00	63.99 ±1.88	64.03 ± 1.55	62.56 ± 1.55	23.78 ± 2.88	36.98 ± 1.47	28.55 ± 2.44	19.56 ± 1.55	63.85 ± 2.45
DOD-raw (%) ^b^	n.d	n.d	n.d	n.d	19.03 ± 0.05	24.47 ± 0.05	24.64 ± 0.05	23.66 ± 0.04	n.d
DOD-blend (%) ^b^	9.39 ± 0.03 ^f^	4.49 ± 0.05	1.95 ± 0.01	1.97 ± 0.04 ^f^	24.06 ± 0.05	18.86 ± 0.05	21.14 ± 0.05	29.17 ± 0.03	n.d
DOD ^h^ (%) ^b^	7.25 ± 0.02	7.15 ± 0.03	1.74 ± 0.02 ^k^	1.53 ± 0.03 ^k^	56.99 ± 0.01	71.45 ± 0.06	78.46 ± 0.04	66.05 ± 0.01	n.d
HHV (MJ/kg)	24.02 ± 0.04	26.66 ± 0.05	26.26 ± 0.02	25.59 ± 0.06	28.23 ± 0.04	28.83 ± 0.08	29.05 ± 0.06	28.86 ± 0.06	21.56 ± 0.06

^b^ wet basis, ^db^ dry basis, ^f^ degree of oxygenation (-), ^k^ hydrous effect, DOD ^h^ degree of dehydration (-), ^ar^ as received, ^a^ determined by difference, and n.d not determined.

## Data Availability

The data will be made available on request.
